# Shoulder Lump

**Published:** 1891-09

**Authors:** Williamson Bryden


### SHOULDER LUMP.

By Williamson Bryden, V. S.

In the April (1890) number of The Journal of Comparative Medicine and Veterinary Archives, I reported a case of what was headed "Fibroid Tumor of the Shoulder."

A heavy buckskin horse used on a single-horse beer-wagon, commenced going lame in his off fore and hind feet, but was kept at work till a large hard "tumor" suddenly appeared a little above the point of the shoulder. On examination of the hind foot I found a very severe bruise that required poulticing and treatment for several days before he was fit to be used. The fore foot was in a bruised condition also, and caused the hoof to be placed unevenly on the ground, at the same time this condition of the extremity caused the animal to carry his shoulder forward in an awkward manner, so that the muscles above the "tumor" in front appeared somewhat wasted, plainly showing the predisposing cause and its history. I then examined the collar which was a fairly good fit and of good quality.

Having had a great many cases of the kind, the condition of the extremities, and especially the defective hoofs from their influence on the position of the shoulder and in causing disturbance to the muscular and connective tissues in front of it, had for a long time led me to suspect them of being an important factor in predisposing to this injury, it is therefore with a good deal of satisfaction that I am able to record a still further history of this interesting case, and as I fear greatly misunderstood disease.

I am led to say this after consulting both recent and older writers on the subject. Bouley is said (by a friend of his) to have remarked that "there is suppuration in ninety-five hundredths (95-100) of known cases." Such a remark needs no comment.

A recent book on "Lameness of Horses" in describing "Cold Abscess," recommends for its treatment such an array of painful operations as ought to satisfy the most enthusiastic of "Doctors." Blisters, explorations for pus, puncturings with red hot irons and other *weapons*, acids, caustics, seatons, excision and similar orthodox measures. Surely it is time that the profession left ruts that carry them to such temporizing expedients, such extremities of cruelty. Professor Williams, too, in the last edition of his "Surgery," page 414, although condemning severe external applications, still adhere to old "heroic" measures.

As soon as the hind foot was sufficiently recovered to be shod, he was set at work, the "tumor" having been kept bathed with warm water to remove a slight œdema caused by some degree of friction to the skin over it.

The collar was then opened, and a little of the padding withdrawn, an extra flat pad was made to fit just above the growth; in this condition he was put at work, but loaded lighter and easier. In a few days (four or five), the lump was found to yield on pressure. On being opened, it discharged about a gill of nice creamy *pus-like* matter. The wound then healed, and he was at work in less than two weeks, quite well.

He worked steadily then till last February, when I was again sent for by the owner, (Mr. Van Nostrand, of Crystal Lake Brewery,) to see the same horse. I found the fore foot twisted and requiring shoeing, a "tumor" nearly half the size of the first having made its appearance. I ordered him sent to the forge to get his foot trimmed and the shoe reset. His hoof was kept soft by stuffing every night, and the shoulder was bathed with an astringent wash every night and morning. He was then kept at easy work for a few days and the lump disappeared entirely, *having become absorbed*.

Again, about the beginning of April, on my return from the examinations at the Veterinary Department, McGill University, Montreal, I found him suffering the same as on its first occurrence, with the exception that he was not so lame behind. I repeated the former treatment and he was as well as ever in a few weeks, its course being the same as in the first.

The conclusions arrived at by me are that the changes in the foot affect the muscles of the shoulder and limb adversely, the weight is thrown on it so that the front of the shoulder being too much advanced, is forced against the collar in such a way that the muscles and connective tissues become bruised and strained, a hard, circumscribed lump or swelling, sometimes of great size, then suddenly presents itself. If the animal is laid off work immediately on its appearance, it is not possible to find a *fully developed* centre containing the pus or pus-like matter, but when kept at work an abscess is rapidly formed, and at the end of a few days it will either have become absorbed, which occasionally happens when small, or be ready to open, or perhaps break. All that is then necessary is simple treatment and the wound will heal very rapidly.

My first impression was that such growths were somewhat similar to the soft corn between the toes in the human subject described by John Hunter long ago. The presence or absence of *matter* in such cases being dependent on the number of days the horse has been kept at work after the lump began to make its appearance. If laid up at once it remains nearly stationary for a long time. If kept at work, with the same or a similar collar—the exciting cause — it must mature within a few days.

It has been contended by some that working the animal with such a shoulder must be very painful and likely to cause balking. Of course it is possible to treat the case in a coarse, brutal way, but there is no necessity for this, and if *stimulating* and *irritating* applications that make the skin tender are kept from it, they will not refuse to pull a moderate load.

My conclusions have not been arrived at from a single case, but from a score within the last five years, every one I have had having recovered so far. I sincerely trust that those veterinarians who have such cases will give a fair trial to this method and report on it, for it has been rather a disappointment to me to find some so hard to convince, that they remain skeptical and continue their old treatment, which they say pays better.

